# Evaluation of 3D Robotic-Guided Exoscopic Visualization in Microneurosurgery

**DOI:** 10.3389/fsurg.2021.791427

**Published:** 2022-02-21

**Authors:** Naureen Keric, Harald Krenzlin, Elena Kurz, Dominik M. A. Wesp, Darius Kalasauskas, Florian Ringel

**Affiliations:** Department of Neurosurgery, University Medical Center, Johannes Gutenberg University of Mainz, Mainz, Germany

**Keywords:** robotic 3D exoscope, microscope, NASA Task Load Index, surgical performance, 5-ALA

## Abstract

**Objective:**

The three-dimensional (3D) exoscope is a novel apparatus introduced in recent years. Although an operating microscope (OM) is customarily used, this novel application offers several advantages. Therefore, this study aimed to determine the feasibility of deploying a robotic-guided 3D-exoscope for microneurosurgery and gauge its subsequent performance.

**Methods:**

The use of a 3D exoscope was compared with that of OM during 16 surgical procedures. Postoperatively, surgeons completed an eight-item Likert-scale satisfaction survey. As a second step, a predefined surgical task was then undertaken by surgeons with varying levels of experience, assessing the time entailed. Two questionnaires, the satisfaction survey and NASA task load index (NASA-TLX), were administered.

**Results:**

During routine procedures, the exoscope proved superior in magnification and ergonomic maintenance, showing inferior image contrast, quality, and illumination. It again ranked higher in magnification and ergonomic maintenance during the suturing task, and the OM excelled in treatment satisfaction and stereoscopic orientation. Workload assessment using the NASA-TLX revealed no difference by modality in the pairwise analysis of all components. At varying levels of experience, beginners bear a significantly higher burden in all principle components than mid-level and expert participants (*p* = 0.0018). Completion times for the suturing task did not differ (*p* = 0.22).

**Conclusion:**

The quality of visualization by 3D exoscope seems adequate for treatment and its ergonomic benefit is superior to that of OM. Although experienced surgeons performed a surgical simulation faster under the OM, no difference was evident in NASA-TLX surveys. The 3D exoscope is an excellent alternative to the OM.

## Introduction

The introduction of the operating microscope (OM) in 1957 was a revolutionary event, impacting the field of neurosurgery for decades (1, 2). As a result, morbidity and mortality were lowered, and certain inoperable tumors became treatable in experienced hands (3). In addition, the intuitive surgical performance enabled by stereoscopic view was improved upon as surgical microscopes continued to advance. Additional techniques, such as fluorescence-guided surgery (for gliomas) and indocyanine green (ICG) angiographic imaging, were subsequent milestones in this regard (4–7). Nevertheless, there are some limitations to OM. For example, the lenses are fixed within the system, requiring the surgeon to move with the microscope to ensure proper views at various angles, and deep illumination may be insufficient.

Although first reported in 1994 (8), exoscopic applications in neurosurgery took many years to materialize (9). An assortment of exoscopes has emerged, presenting some advantages and disadvantages relative to OM (10). A wider field of view is considered a distinct advantage of the exoscope, whereas its 2D video telescope operating monitor (VITOM) has proven problematic. The inherent lack of stereopsis impedes coordination of hand and eye and is the most notorious feature (11, 12). However, novel 3D exoscopes have since been developed. The ORBEYE (Olympus, Tokyo, Japan), launched in 2017, is equipped with a camera and a pneumatic arm set on a portable base to provide high-definition 3D images (13–15). After a certain learning curve, the use of this system seemed reasonable in various surgical settings (13–15).

On the other hand, this novel technique is still evolving, and challenges experienced surgeons in transitioning from a familiar OM to 3D visual mode. The AEOS 3D exoscope system (Aesculap Inc, Tuttlingen, Germany) recently released for microneurosurgery has a robotic-guided arm. Another earlier prototype has shown similarities to standard OM in a cadaveric study (16). In the present study, we aimed to evaluate the imaging generated by this robotic-guided 3D exoscope in cranial and spinal surgery and to gauge its surgical performance relative to OM.

## Materials and Methods

### The 3D Robotic-Guided Exoscope

**Figure 1:**
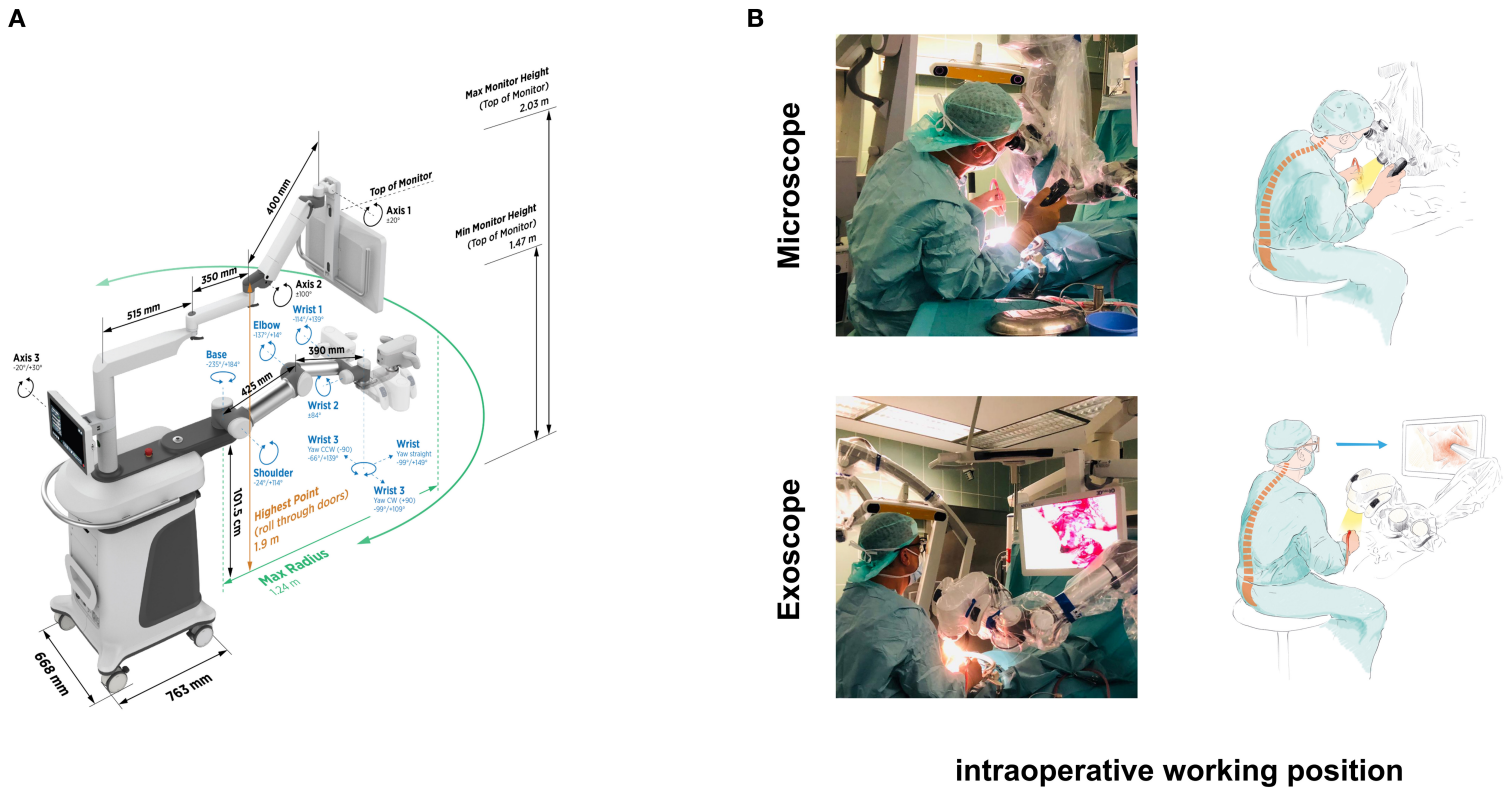
Image of the AEOS exoscope with metric details **(A)**. Sitting work posture using the operating microscope and the exoscope. The posture has to be aligned to match the intended working trajectory using the microscope. At the same time, the surgeon can maintain an upright and more natural posture while looking at the HD monitor attached to the exoscope **(B)**.

The Aesculap^®^ AEOS DSM (Digital Surgical Microscope) (Aesculap Inc, Tuttlingen, Germany) is a novel pre-production 3D exoscope equipped with a robotic-guided arm. The robotic arm comprises six motorized joints with a total shaft length of 81.5 cm. The digital movement velocity is infinitely variable adjustable. In contrast to other exoscopic systems, the robotic arm is a unique characteristic of the AEOS DSM, offering similar capabilities to conventional OMs. The camera is controlled using handles with buttons, which can be assigned individually with different commands and functions. The optimal distance to the region of interest is between 20 and 45 cm. The field of view ranges from 70 × 130 mm (minimum zoom) to 5 × 9 mm (maximum zoom). The corpus dimensions are 82.6 cm (length) × 122.7 cm (width) × 193.8 cm (height). The system is equipped with a 3D 4K Monitor, which can be positioned variably according to procedure requirements and OR setup. Our system included a deep ultraviolet (DUV 400) and infra-red (DIR 800) modules to enable visualization of 5-aminolevulinic acid (5-ALA) and Indocyanine Green Angiography (ICG) (Figure 1).

### Intraoperative Evaluation

A total of 16 operations conducted by our neurosurgical staff deployed the AEOS 3D exoscope. A conventional OM (OPMI Pentero; Carl Zeiss Meditec AG, Jena, Germany) was available at any time for comparison purposes or those dissatisfied with the exoscope. Operative time and participant satisfaction were assessed through a Likert-scale survey addressing image quality, contrast, illumination, magnification, visual field, ergonomic maintenance, and stereoscopic orientation. These were rated as very satisfied, somewhat satisfied, neither satisfied nor dissatisfied, somewhat dissatisfied, or very dissatisfied.

### Predefined Surgical Task and Participants

As a second step, a predefined surgical task was performed by active surgeons or medical students in their final year of schooling. The 28 participants were grouped by level of surgical experience as follows: inexperienced (medical students, 7), beginners (new residents, 7), mid-level acumen (residents in final 2 years of training, 8), and experts (consultants and chief surgeon, 8). The order of instrumentation used (OM vs. 3D exoscope) was randomly assigned. OM and exoscope settings were identical for all participants, each having consented to participate in an analysis of anonymized data. (characteristics of the study group are displayed in Table 1).

**Table 1:** Characteristics of the study group.

	**Students**	**Beginner**	**Experienced**	**Expert**	**Total**
Number of participants	6	7	8	6	27
Age					
Sex (female/male)	4/2	2/5	0/8	1/5	
Right-hand-dominance (%)					
Surgical training (years)	0	2.1 ± 2.1	8 ± 1.7	16.1 ± 3.7	

The stipulated task was to suture a 10-cm linear skin incision of the chicken thigh, using single button sutures in separate runs by OM and exoscope, or vice versa. The chicken thigh was fixed onto the operative table. For suturing, we supplied 4-0 polyester material (Ethibond EXCEL; Ethicon [Johnson & Johnson], Somerville, NJ, USA). Each participant received surgical forceps, a needle holder, and a pair of scissors. All instrumental work was performed individually. Suture points were precisely marked (1 cm apart), starting and ending 0.5 cm from the cut edge. Three instrumental knots were required. A third person recorded elapsed times from the first insertion to the laydown of instruments.

The primary study endpoint was performance quality, comparing outcomes *via* OM or exoscope in surgeons and medical students. Secondary endpoints were satisfaction with and manageability of the two instruments. The National Aeronautics and Space Administration Task Load Index (NASA-TLX) provides a widely accepted and validated multidimensional tool containing six predefined dimensions (three task-imposed dimensions: mental, physical, and temporal demands, three coping dimensions: self-rated performance, effort, and frustration level) to measure overall workload after completing a task (17). Immediately after the performance, the NASA Task Load Index (NASA-TLX) was recorded from every single test person. Once both tasks were completed, participants were independently and anonymously surveyed using the above Likert-scale questionnaire and the NASA-TLX.

### Statistical Analysis

At every experience level, performance and satisfaction were analyzed, expressing data as mean±standard deviation (SD) values. Students *t*-test (two-stage step-up method of Benjamini, Krieger, and Yekutieli) was applied. Outcome parameters were subjected to two-way analysis of variance (ANOVA), using Tukey's multiple comparison *post hoc* test. Standard software (Prism v8.4.2 for macOS; GraphPad Software, San Diego, CA, USA) was engaged, setting significance at *p* < 0.05.

## Results

### Intraoperative Evaluation

**Figure 2:**
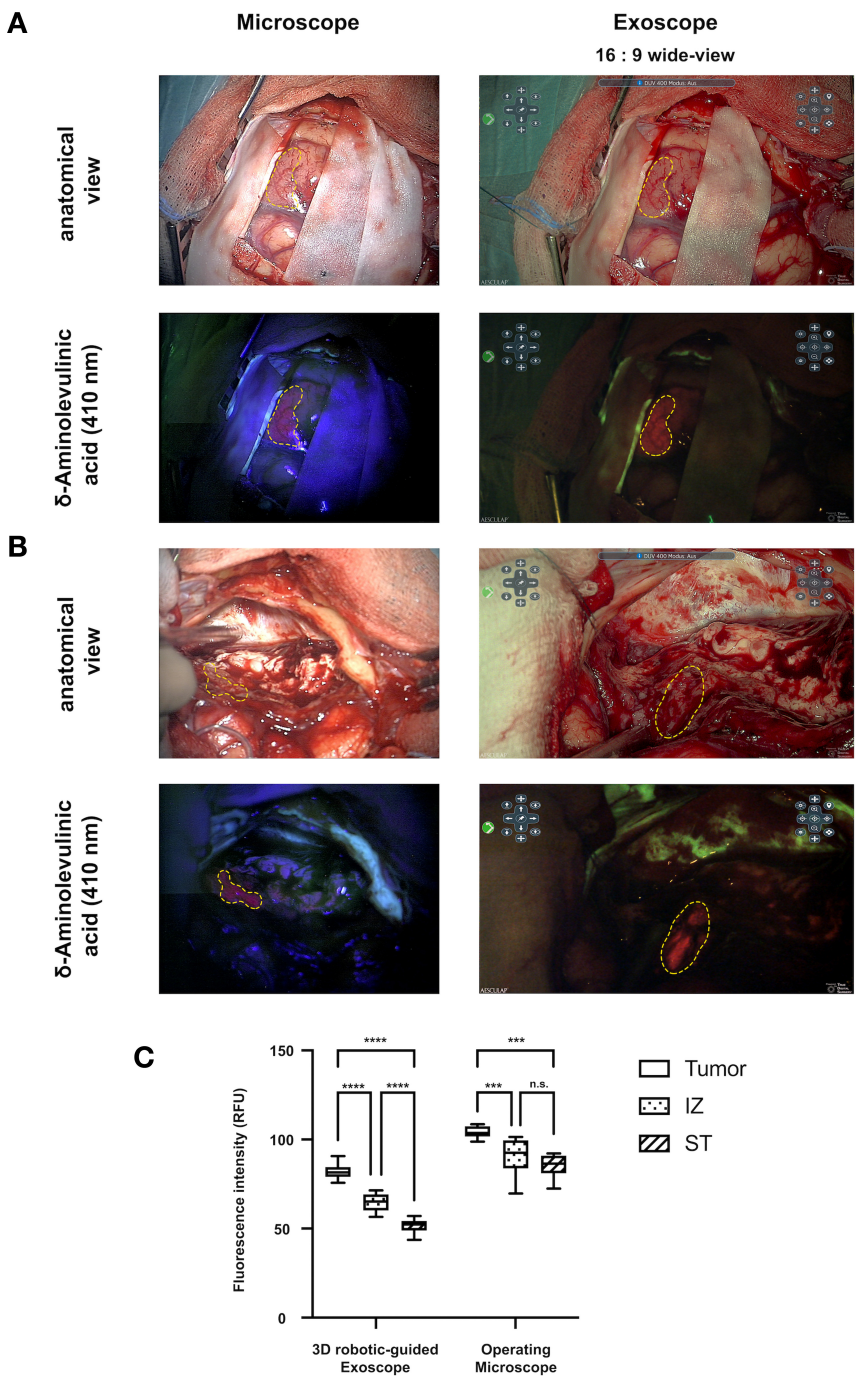
**(A)** Side-by-side comparison of work views using the operating microscope and the exoscope. **(B)** Smaller (two upper rows) and higher (bottom rows) magnification using reflected light and 5-aminolevulinic acid. The picture ratio cor responds to the actual screen size. The dotted line indicates the tumor margin. **(C)** Mean 5-Ala fluorescence was higher in OM images compared to those from the exoscope. (***P < 0.005, ****P < 0.0001).

**Figure 3:**
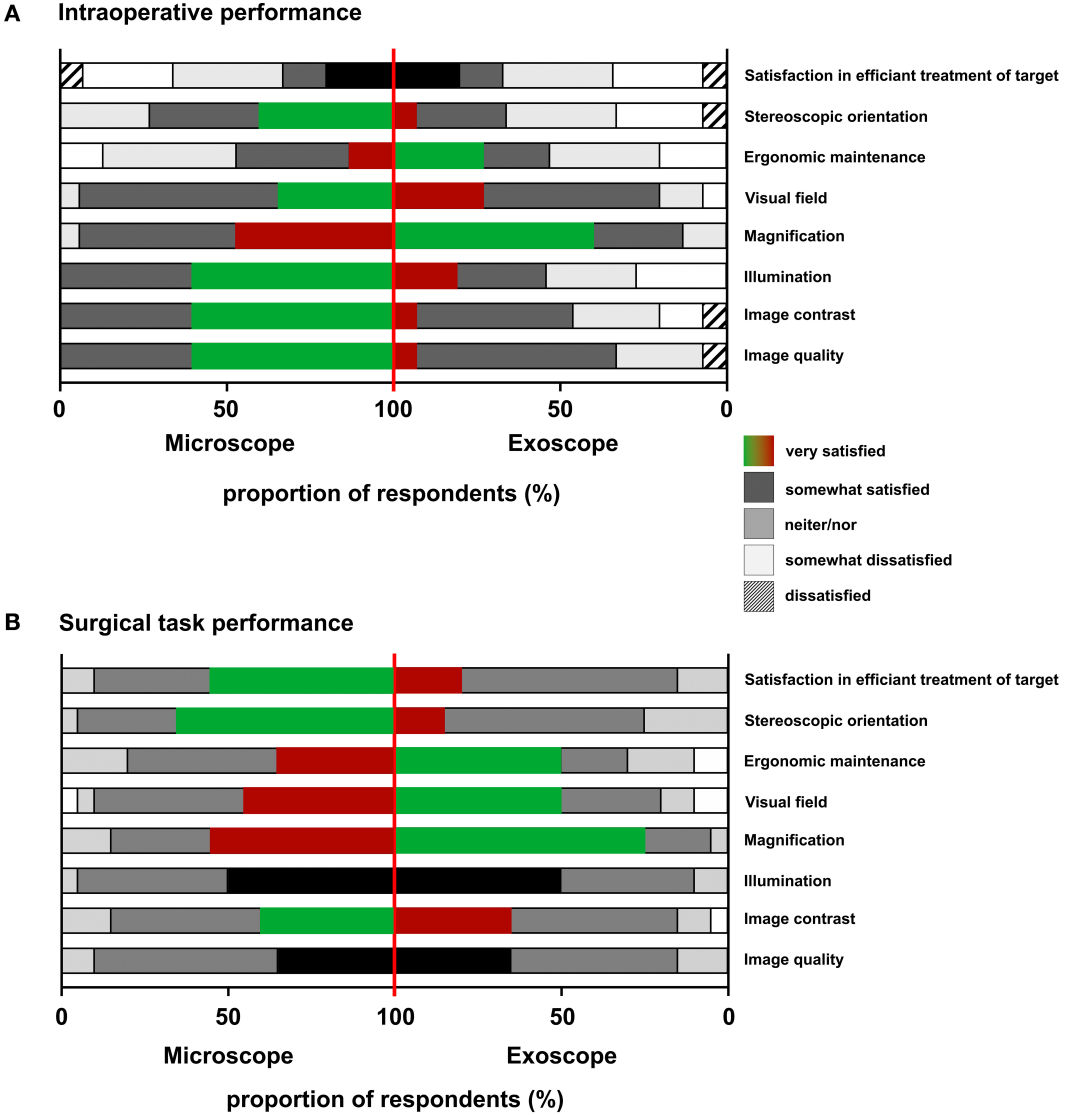
Standardized evaluation using an eight-item questionnaire. The exoscope remained advantageous in ergonomic maintenance and magnification in routine procedures, while the microscope performed superior in contrast, quality, and illumination **(A)**. Using the surgical microscope, higher treatment satisfaction and better stereoscopic orientation were stated, while the exoscope performed better in magnification and ergonomic maintenance during the standardized suturing task **(B)**.

Twelve cranial and four spinal operations were performed using the exoscope. During three operations (cranial), the surgeon decided to switch due to insufficient image contrast, depth of field, and tissue differentiation; in one case, the surgeon switched to the OM due to reasons of unfamiliarity; and in seven cases, the surgeons opted to switch to the OM and back to the exoscope for purposes of comparison. (details are displayed in Table 2). Fluorescence imaging was applied via the exoscope in four procedures (three gliomas and one aneurysm clipping) and was perceived to be similar or superior when compared to the OM by the respective surgeons (Figure 2). Mean 5-Ala fluorescence acquired from 10 ROIs within either tumor, infiltration zone (IZ) or non-fluorescent soft tissue (ST) was higher in images taken from the OM (tumor: 103.98 ± 3.07; IZ: 91.02 ± 9.77; ST: 85.24 ± 6.58; *p* < 0.0001) compared to those originating from the exoscope (tumor: 81.94 ± 4.51; IZ: 64.44 ± 5.22; ST: 51.69 ± 4.17). For both instrument statistically significant differences were found between tumor tissue, IZ and ST. To assess AEOS exoscope deployment during routine neurosurgical procedures, surgeons were surveyed using an eight-item Likert-scale questionnaire. Treatment satisfaction scores that were generated did not differ by modality. The exoscope proved advantageous in terms of ergonomic maintenance and magnification; OM perceived better at image contrast, image quality, and illumination (Figure 3A).

**Table 2:** Operative cases.

	**Case no**.	**Diagnosis**	**Switch (assessment)**
Spinal tumor	1	Sacral spinal neurinoma	No
	2	Paraspinal thoracic tumor	No
Degenerative spine	1	Lumbar spinal stenosis	No
	2	Cervical stenosis, corpectomy C5	Yes (unfamiliar with exoscope)
Cranial tumor	1	Temporal left sided glioblastoma	Yes (poor tissue differentiation)
	2	Occipital metastasis	Yes (poor visualization)
	3	Postcentral metastasis	Yes (exoscope malfunction)
	4	Fronto-temporal epidermoid cyst	Yes (comparison to OM)
	5	Recurrent meningioma middle fossa	Yes (comparison to OM)
	6	Parietal metastasis	Yes (unsatisfied with depth of field)
	7	Temporal right sided glioblastoma	Yes (comparison to OM)
	8	Occipital metastasis	Yes (comparison to OM)
	9	Frontal glioblastoma	Yes (comparison to OM)
	10	Posterior fossa ependymoma	Yes (comparison to OM)
Cranial vascular	1	Medial cerebral artery aneurysm	Yes (comparison to OM)
Trauma	1	Anterior skull base flap	No

### Predefined Surgical Task

None of the participants had previously used the AEOS exoscope. The various groups were determined by prior neurosurgical OM experience. After the suturing task, treatment satisfaction, and stereoscopic orientation for OM use, the exoscope excelling again in magnification and ergonomic maintenance (Figure 3B).

**Figure 4:**
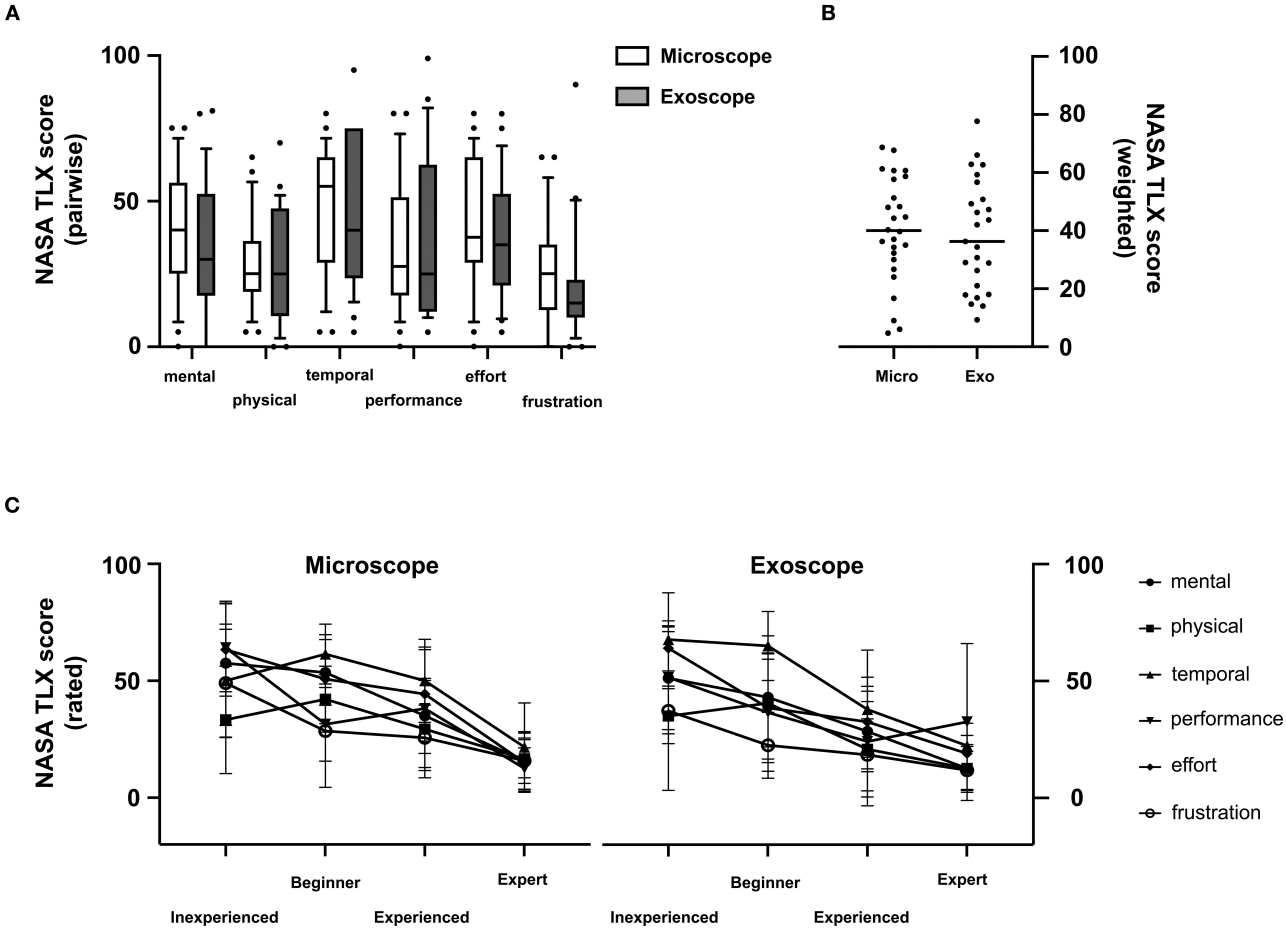
Multidimensional perceived workload assessment using the NASA-TLX. Pairwise analysis of all principle components (mental, physical, temporal, performance, effort, frustration) **(A)** and overall weighted workload scores **(B)** showed no statistically significant differences across all participants. The principle components were perceived as less demanding by experienced compared to inexperienced surgeons **(C)**.

Analysis of the six scored items in NASA-TLX surveys (Figure 4A) indicated no significant differences among participants by modality. OM ratings were as follows: mental demand, 38.46 ± 21.0; physical demand, 28.85 ± 16 51; temporal demand, 47.89 ± 22.19; performance, 34.62 ± 23.58; effort, 41.92 ± 22.94; and frustration, 25.77 ± 19.01. Exoscope ratings were similar, recorded as follows: mental demand, 33.28 ± 23.62; physical demand, 27.24 ± 19 0; temporal demand, 47.72 ± 25.73; performance, 35.12 ± 27.81; effort, 37.28 ± 21.49; and frustration, 21.76 ± 19.81. Subgroup analyses at core experience levels also showed no significant differences by method (OM vs. exoscope).

Overall subjective NASA-TLX workload scores for OM and exoscope were 40.15 ± 18.38 and 38.48 ± 19.08, respectively (Figure 4B). Instrument usage did not differ significantly for participants as a whole or in subgroups.

Analyses of scored items by experience level showed significantly greater burdens of all components (except frustration) in beginners, compared with mid-level and expert participants (*p* = 0.0018). Although effort (*p* = 0.0067), temporal demand (*p* = 0.016), and performance (*p* = 0.0047) weighed more heavily on mid-level (vs. expert) participants, mental demand, physical demand, and frustration were not rated differently. Using the exoscope, mental (*p* = 0.017), physical (*p* = 0.03), and temporal (*p* = 0.03) demand were significantly more troublesome for inexperienced (vs. experienced) participants, mid-level and expert participants showing similarities (Figure 4C).

**Figure 5:**
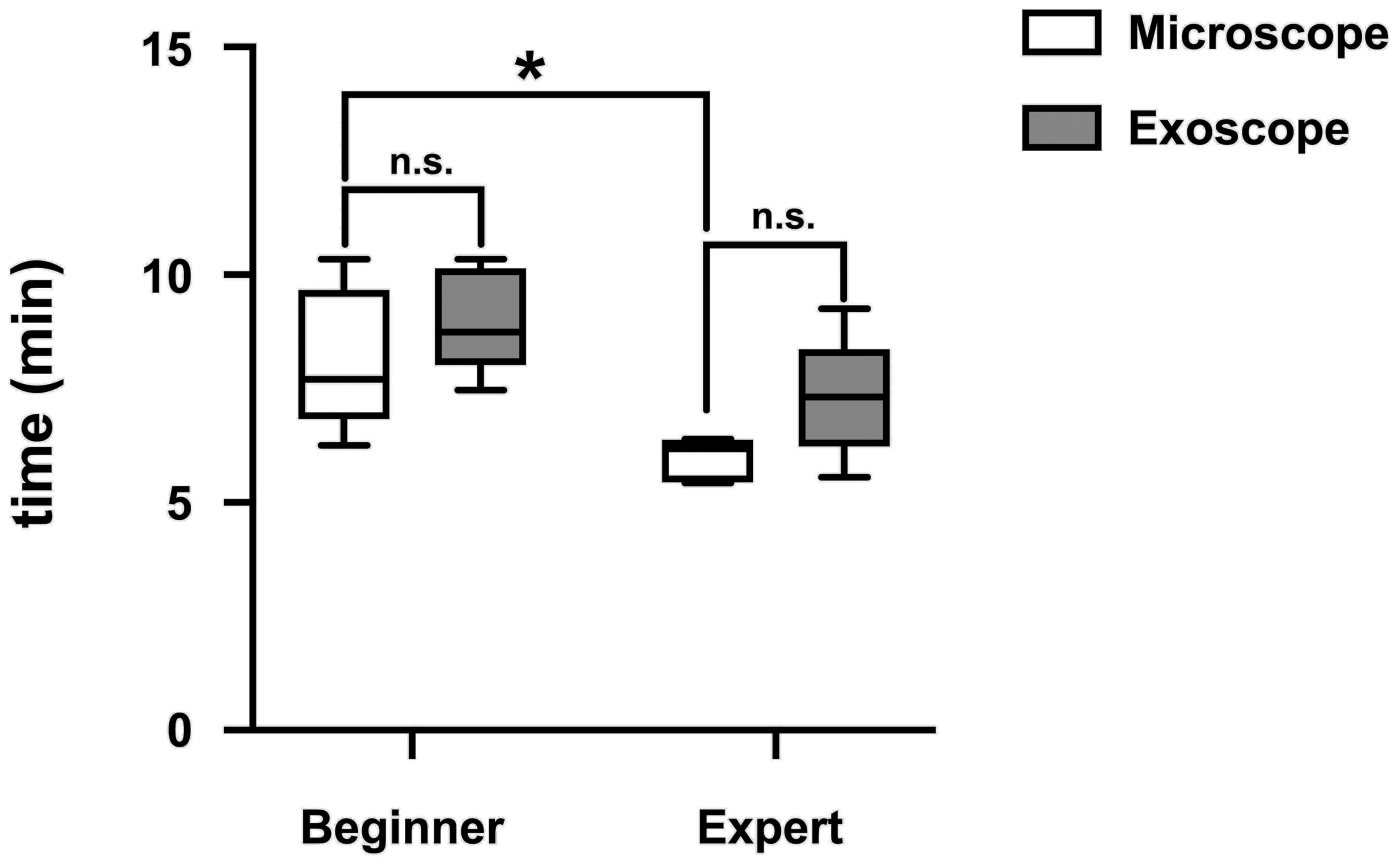
Experienced surgeons had a statistically significant shorter task completion time compared to inexperienced surgeons. No difference was found between both modalities. (*P < 0.05).

Task completion times for OM and exoscope did not differ significantly. Subgroup analyses likewise showed no significant differences in modalities by experience level. Task completion by OM was significantly longer for those with no (vs. longstanding) surgical experience (9.29 ± 3.5 min vs. 6.0 ± 0.45 min; *p* = 0.01), but no significant difference was recorded for the exoscope (*p* = 0.05) (Figure 5). Subjective NASA-TLX workload scores significantly and negatively correlated with task completion time for each modality.

## Discussion

Since the introduction and establishment of the OM by Kurze in 1957, such systems have been refined and further evolving (1, 5, 6). The principles of stereoscopy, visual field, and 3D visuals/perceptions have guided the development. Neurosurgical visualization by OM, endoscopy, and now *via* exoscopic systems is subject to rapid change. The most recent generation of exoscopes involving 2D and high-definition 3D systems has become an alternative to conventional OM. Herein, we assessed the visualization potential, feasibility, and efficacy of a robotic-guided 3D exoscope during cranial and spinal procedures. We also gauged its performance (relative to OM) in a predefined surgical simulation.

The exoscope used in our study is a pre-production model designed by Aesculap (Tuttlingen, Germany) to facilitate an easier transition from convention OMs to a 3D digital microscope. The ergonomic design resembles hallmarks of the OM, such as a handle-controlled camera paired with a modern robotic arm for optimized ergonomic properties. So far, the AEOS exoscope has not been used in man. Our study provides the first insight into the performance of the AEOS system during routine clinical cases adding the dimension of time and pressure to evaluate the novel exoscopic system.

### Intraoperative Application

Our staff performed several cranial and spinal procedures. In ~40% of the operations, the surgeons opted to switch from OM for purposes of comparison (primarily). In other studies, various operative procedures have been conducted using the exoscope, although no reports have yet presented any reasons to abandon conventional OM (18).

Four fluorescence-guided operations were performed using the 3D exoscope, switching between exoscope and OM in one procedure. The fluorescence of 5-ALA was subsequently much more intense in color and clearer in contrast. An overlay mode of the area with active 5-ALA fluorescence and the white light view is an additional function of the 3D exoscope—this aids in identifying and resecting tumors. In a study by Piquer et al. 30 patients underwent fluorescence-guided tumor resections, and eight had image-guided biopsies. The biopsy material was examined in fluorescence mode of the exoscope (HD Xoscope, HDXO-SCOPE; Karl Storz Endoscopy, Tuttlingen, Germany) (19). In addition, one aneurysm clipping was performed *via* exoscope. The use of ICG angiography to confirm sufficient clipping by exoscope showed complete occlusion of the aneurysm, providing excellent image quality and contrast. Exoscope based ICG (ICG-E) has been used in only a few cases so far but proved to be safe and feasible. Our findings confirm the advantages of ICG-E (20).

One critical issue raised by surgeons was image contrast and tissue differentiation capability, especially when distinguishing between glioma or brain metastasis and normal brain tissue. In three cases, the surgeons switched to the OM due to insufficient visualization. This issue may perhaps be improved through software changes by the company.

In comparing the robotic-guided arm of the exoscope to that of OM, its movements were softer and somewhat slower but more precise. Installation in the operating room was similar to that of the OM, although always positioned at the side of the lesion. The 3D monitor was placed directly in front of surgeons (Figure 1B). Due to this setup, both chief and assisting surgeons (wearing 3D glasses) shared the same view, facilitating co-surgical interaction. The exoscope rating by eight-item Likert-scale survey was superior in terms of ergonomic maintenance and magnification. However, surgeons still scored image contrast, quality, and illumination better for the OM. Our findings fall in line with previous reports by Muhammad et al. where depth perception was inferior using an exoscope despite a higher image quality in a series of eight routine neurosurgical procedures (21). The perceived advantages and downsides of the exoscope in neurosurgical practice not only hold true in comparison to OMs but also in comparison to neuroendoscopes (22). Ergonomic advantages offered by the AEOS exoscope were highlighted by certain cases where a direct line of vision between the OMs and the operating field was hard to establish (e.g., lesions of the occipital lobe and posterior fossa done in supine position due to medical requirements of the patient). Here, a viewer independent position of the exoscope camera could be realized, allowing for a much more convenient posture of the surgeon.

### Surgical Task and Task Load Index

Our comparative study among medicals students and surgeons at various stages of experience level showed no difference in time required for task completion using OM or exoscope, regardless of the order undertaken. In other exoscopic systems, operative and handling times were much longer than those of the OM, the lack of stereoscopic vision primarily culpable (10, 23). Hence, the 3D system applied herein yields sufficient stereoscopic visualization for intuitive handling. The workload for task completion assessed by NASA-TLX surveys was higher in younger and inexperienced participants using both systems.

In a separate study investigating a 2D exoscopic system, the task load among inexperienced participants was also higher, compared with an OM approach (9, 24). Again, the most critical factor was the lack of stereoscopic vision. Unlike our study, the overall workloads of OM and exoscope did not differ, although times for task completion by OM or exoscope were similar. Ultimately, we found that higher-level experience led to faster task completion in both systems, proving that practice improves skills (25). In the eight-item scoring of participants, OM ranked higher in treatment satisfaction and stereoscopic orientation; the exoscope rated better in magnification and ergonomic maintenance. Reliance on OM during surgery requires access to certain operative angles and slim keyhole corridors, imposing uncomfortable head, neck, and back posturing on surgeons (Figure 1). It is thus not surprising that an ergonomic work posture is one of the most acclaimed assets of this new visualization system in many studies (14–16, 26–28).

## Conclusion

Since the introduction of the OM, it has become an indispensable fixture of the operation room. It has been developed continuously but not fundamentally and shows some improvable deficiencies. Herein, we investigated the quality of visualization enabled by a novel 3D exoscopic system, comparing its performance with conventional OM during neurosurgical procedures and in a predefined surgical simulation. This study confirms that an exoscopic system is an excellent alternative to the OM, applicable in all types (even fluorescence-guided) of neurosurgical operations. Its most significant advantage is ergonomics, allowing a physiologic posturing of surgeons. However, the most critical limitations were tissue differentiation and image contrast, both surpassed by OM utilization. During a stipulated suturing task, three times required for completion did not differ by modality, and no increased task load was conferred due to system unfamiliarity. Assuming some technical improvements can be made, the exoscopic system seems a reasonable adjunct or alternative to conventional OM.

## Data Availability Statement

The original contributions presented in the study are included in the article/supplementary material, further inquiries can be directed to the corresponding author.

## Ethics Statement

Patient data acquisition and analysis were performed retrospectively and anonymously. According to the local laws of Rhineland Palatinate, no informed consent or formal approval of the Ethics Committees of the medical association of Rhineland Palatinate, Germany, is necessary for such kind of analysis.

## Author Contributions

NK and FR: conceptualization and validation. NK, HK, and FR: methodology. HK and NK: software. NK, HK, DK, and DW: formal analysis. NK, EK, HK, and DW: data curation. NK and HK: writing—original draft preparation and visualization. DK, FR, EK, and DW: writing—review and editing. FR: supervision. All authors have read and agreed to the current version of the manuscript.

## Conflict of Interest

The authors declare that the research was conducted in the absence of any commercial or financial relationships that could be construed as a potential conflict of interest.

## Publisher's Note

All claims expressed in this article are solely those of the authors and do not necessarily represent those of their affiliated organizations, or those of the publisher, the editors and the reviewers. Any product that may be evaluated in this article, or claim that may be made by its manufacturer, is not guaranteed or endorsed by the publisher.
